# Imaging Flow Cytometry: Development, Present Applications, and Future Challenges

**DOI:** 10.3390/mps7020028

**Published:** 2024-03-23

**Authors:** Savvas Dimitriadis, Lefkothea Dova, Ioannis Kotsianidis, Eleftheria Hatzimichael, Eleni Kapsali, Georgios S. Markopoulos

**Affiliations:** 1 Hematology Laboratory, Unit of Molecular Biology and Translational Flow Cytometry, University Hospital of Ioannina, 45100 Ioannina, Greece; sdimitr@uoi.gr (S.D.); ldova@uoi.gr (L.D.); 2 Department of Hematology, University Hospital of Alexandroupolis, Democritus University of Thrace, 69100 Alexandroupolis, Greece; ikotsian@med.duth.gr; 3 Department of Hematology, Faculty of Medicine, University of Ioannina, 45110 Ioannina, Greece; ehatzim@uoi.gr (E.H.); ekapsali@uoi.gr (E.K.); 4 Department of Surgery, Faculty of Medicine, University of Ioannina, 45110 Ioannina, Greece

**Keywords:** flow cytometry, imaging flow cytometry, artificial intelligence, phenotype analysis

## Abstract

Imaging flow cytometry (ImFC) represents a significant technological advancement in the field of cytometry, effectively merging the high-throughput capabilities of flow analysis with the detailed imaging characteristics of microscopy. In our comprehensive review, we adopt a historical perspective to chart the development of ImFC, highlighting its origins and current state of the art and forecasting potential future advancements. The genesis of ImFC stemmed from merging the hydraulic system of a flow cytometer with advanced camera technology. This synergistic coupling facilitates the morphological analysis of cell populations at a high-throughput scale, effectively evolving the landscape of cytometry. Nevertheless, ImFC’s implementation has encountered hurdles, particularly in developing software capable of managing its sophisticated data acquisition and analysis needs. The scale and complexity of the data generated by ImFC necessitate the creation of novel analytical tools that can effectively manage and interpret these data, thus allowing us to unlock the full potential of ImFC. Notably, artificial intelligence (AI) algorithms have begun to be applied to ImFC, offering promise for enhancing its analytical capabilities. The adaptability and learning capacity of AI may prove to be essential in knowledge mining from the high-dimensional data produced by ImFC, potentially enabling more accurate analyses. Looking forward, we project that ImFC may become an indispensable tool, not only in research laboratories, but also in clinical settings. Given the unique combination of high-throughput cytometry and detailed imaging offered by ImFC, we foresee a critical role for this technology in the next generation of scientific research and diagnostics. As such, we encourage both current and future scientists to consider the integration of ImFC as an addition to their research toolkit and clinical diagnostic routine.

## 1. Introduction

Flow cytometry (FC) is a powerful methodology for the characterization of complex phenotypes in cellular populations, as well as the quantification of cellular processes such as proliferation, cell death, and cell differentiation [1]. The rationale of FC is based on the analysis of the spectral characteristics of cells in a homogeneous liquid mixture. A standard flow cytometer, in which FC analysis is performed, can be divided into three distinct systems: a hydraulics system, an optical system, and an electronics system. The hydraulics system performs hydrodynamic focusing in order for cells to pass sequentially, as single events, through an interrogation point. At this point, as part of the optical system, a laser source is used for excitation-specific fluorophores (that have been added in a preanalytical step), while the emitted fluorescence from each cell is passed through detection filters. Third, the electronics system stores fluorescence signals in a digital format [1,2,3,4]. These characteristics make flow cytometry invaluable in several scientific fields such as hematology, immunology, and oncology, among others [5,6,7,8,9,10,11,12,13,14,15].

**Figure 1:**
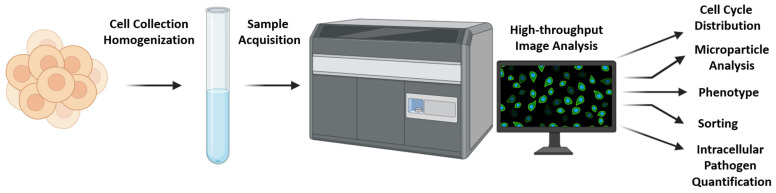
The workflow for imaging flow cytometry (ImFC), a technology that combines flow cytometry and microscopy to enable high-throughput analysis of cells. Cells are collected and homogenized to ensure a consistent sample containing single cells, which is crucial for accurate flow cytometry. Cell suspension is then loaded into the ImFC instrument (an Imagestream MK II is depicted for reference), where individual cells are isolated and aligned for imaging. As cells pass through the ImFC device, they are imaged at high speed, capturing detailed fluorescence and brightfield images for each cell. The collected images are analyzed to extract multiple data points. The technology has several applications, including assessment of cell cycle stages based on nuclear DNA content and morphology; detection and characterization of small particles like exosomes or microvesicles that may be present with the cells; morphological and phenotypic profiling of cells, including size, shape, and internal complexity; sorting capabilities to physically separate and collect cells based on the analysis (not available in the depicted cytometer); and quantitative analysis of pathogens inside cells, which is vital for studying infections and immune responses. ImFC workflow enables rapid and detailed cellular analysis, facilitating advanced research in cell biology, immunology, and related fields.

Imaging flow cytometry (ImFC) stands as an innovative and technologically advanced offshoot of conventional flow cytometry (FC), distinguished by new modifications and improvements to existing techniques. Using a camera, ImFC is able to provide granular and high-quality visual information about the detected cells. More specifically, it provides broad information about the morphology, or structural characteristics, of the cell. This includes a detailed analysis of the cell size, shape, internal structure, and the distribution of specific components or markers within the cell. Thus, the combination of imaging technologies with flow cytometry represents a major advance in cellular analysis [16]. This way, users have a supplementary source of information to validate their fluorescence data and provide a comprehensive and accurate characterization of cell populations. The principles and key applications of ImFC are presented in Figure 1.

In the evolving landscape of cytometric technologies, ImFC emerges as a cornerstone advancement, distinguishing itself by its application versatility, particularly in the study of non-adherent cells, and its unmatched capability in detecting rare cell populations. As such, ImFC has the advantages of both flow cytometry and microscopy. First, it is advantageous over typical FC in a manner that provides structural information apart from the spectral properties of cells. ImFC’s ability to capture high-resolution images of cells in flow enables the visualization of cellular morphology, the organization of intracellular components, and the spatial relationships between different cell markers. This is a significant advancement over conventional FC, which is largely limited to measuring fluorescence intensity without providing any contextual imagery of the cellular structures producing these signals. By integrating structural with spectral data, ImFC facilitates a more nuanced understanding of cellular behavior, phenotypic variations, and complex biological processes [17]. Second, it provides a high throughput, a feature that is not available in fluorescence microscopy [18]. Unlike traditional microscopy, IFC transcends conventional limitations by integrating the ability to analyze an extensive array of markers simultaneously and in a high throughput. The technological nuances that position ImFC as a revolutionary tool in cytometry include its robust data acquisition, uniform field illumination, and the automated segmentation of images, relying less on supervised analysis than microscopy [19]. These technological strides not only enhance the precision and efficiency of cellular analysis, but also pave the way for groundbreaking research in cellular biology and contribute to the scientific community’s understanding and exploration of cellular dynamics [17].

The current review unfolds the chronological progression of imaging flow cytometry (ImFC), from its historical roots to its contemporary applications, and looks forward to the potential challenges in its future deployment. It also critically addresses the pivotal steps required to transition this technology from laboratory research to practical clinical use.

## 2. History of Imaging Flow Cytometry

FC development through the years is based on the same principles as a fluorescence microscope, with the main difference being that the fluorescence is digitalized as a signal and not as an image [1]. Following the vision that a cytometer could employ imaging capabilities, an initial implementation of ImFC was developed as early as 1978 by a research team at Rochester University, who envisioned that a cytometer could utilize imaging capabilities, leading to the initial development of imaging flow cytometry [20,21]. The authors developed a slit-scan flow system that performed slit-imaging. This was a preliminary technological milestone that was, however, ahead of its time, as it needed necessary technological evolution that would take more than 20 years. Critical technology implementations in detectors and imaging systems led to the first commercially available imaging flow cytometers [22]. Commercial systems, even though they lacked resolution compared to fluorescence microscopes, allowed for the analysis of protein particles [23]. These implementations, though promising, were never commercialized and the field remained hindered for nearly two decades, mainly because new technologies were in need to support ImFC requirements.

For the next two decades, the scientific and technological community worked at refining and enhancing this innovative technique. During this period, the major hurdles stemmed from the limitations of then-existing technology in detectors and imaging systems. The process of capturing and interpreting detailed cell images in real time at the scale needed for flow cytometry required far more sophisticated and powerful hardware and software than those available at that time. Throughout the 1980s and 1990s, significant efforts were made to develop the necessary technological components and increase their resolution and speed while ensuring their reliability. By the turn of the century, research and development in the field began to bear fruit, ultimately leading to the launch of the first commercially available imaging flow cytometers, along with several other developments in the field of cytometry. The advancements in creating these ImFC instruments significantly enhanced its capabilities. These include the introduction of high-resolution analysis with a 60× objective, variable objectives for tailored throughput options, the ability to switch between flow cytometry and ImFC modes, and an increased laser line versatility for better reagent compatibility. These improvements position ImFC uniquely in biomedical research, especially for imaging cells in suspension, offering advantages over systems like high-throughput microscopy designed for adherent cells [18]. ImFC instruments marked the beginning of ImFC in cellular analysis, in parallel to multiparameter flow cytometry [1,24,25,26,27].

## 3. State of the Art: Current Imaging Cytometers and Present Applications

The trajectory of imaging flow cytometry (ImFC) saw a marked progression with the first successful introduction of commercial ImFC systems, notably Imagestream and FlowSight, originally developed by Amnis Corporation (and currently under Cytek Biosciences). These robust systems revolutionized the field with their superior capabilities and advanced features. These intellectual properties were acquired by EMD Millipore in 2011. As of the present day, these systems are marketed and commercialized under the umbrella of the Luminex Corporation group. This consolidation represents an important milestone in the commercial trajectory of ImFC systems, highlighting their growing prominence in the scientific and medical industries. A number of key applications of imaging flow cytometry are summarized in Table 1.

Headland et al. showed that the ImageStream Mk II imaging flow cytometer demonstrates remarkable capabilities in the analysis of microparticles and calibration beads, ranging from 20 nm to 1 µm. It excels in minimal sample preparation and volume requirements, enabling accurate quantification in various samples, including whole blood and plasma. Significantly, it outperforms traditional cytometers like the BD LSRFortessa, detecting nanoparticles as small as 20 nm, showcasing a superior sensitivity. With its advanced imaging in multiple wavelengths, including brightfield images and fluidics control, it achieves a high correlation in quantifying microparticles, offering a breakthrough in microparticle research through detailed phenotypic analyses and real-time generation kinetics [49]. Keeping with this, Erdbrugger et al. confirmed that ImFC, using an Imagestream imaging FC, represents a more sensitive method for characterizing microparticles (MPs) than traditional FCM alone. This approach overcomes some limitations of conventional FCM by providing morphological insights and allowing for the differentiation between true single events and aggregates or debris, despite challenges in detecting MPs smaller than 0.200 µm and the need for better standardized calibrators [50]. Given the performance and high resolution of traditional FC in the quantitative and qualitative analysis of nano-sized vesicles from cells (up to 100 nm) [51], ImFC has the prospect to be suitable for nanobiology studies and clinical applications like analyzing vesicle-based biomarkers.

A very interesting application is the label-free analysis of cell cycle distribution [52]. Utilizing high-throughput ImFC, researchers showcased a polarized distribution of antigens in B cells throughout an immune response, a characteristic maintained across descendants. As B cells trigger humoral immune responses, they gather antigen to present to corresponding T cells. Following antigen accumulation by mouse B cells, its polarized arrangement persists for prolonged durations in vivo. High-throughput ImFC revealed that this polarization remains intact through B cell mitosis, leading to uneven antigen distribution among offspring [41]. In conclusion, high-throughput ImFC successfully revealed the cell cycle distribution of cells, as well as a consistent pattern of polarized antigen distribution in B cells during immune responses, a pattern that persists across generations. Thus, this technological advancement plays a pivotal role in enhancing our understanding of the mechanics of immune response activation and its implications for B cell division and subsequent antigen segregation among progeny.

Another innovative approach presented a sheathless, microfluidic imaging flow cytometer that incorporated stroboscopic illumination for blur-free fluorescence detection at an ultra-high analytical throughput, enabling the detection of the internal localization of P-bodies and stress granules. This system was capable of multiparametric fluorescence quantification and sub-cellular localization down to 500 nm with microscopy image quality, achieving analytical throughputs in excess of 60,000 and 400,000 cells per second for fluorescence and bright-field detection, respectively [53].

ImFC has been applied in the analysis of intracellular pathogens. Haridas et al. reported an Imagestream-based analysis of Toxoplasma gondii and Mycobacterium tuberculosis infections in cell lines [42]. Phanse et al. described an ImFC method for analyzing the internalization of Salmonella species [54]. Also, studies have shown the analysis of malaria development [55]. This type of analysis offers a prospect for studying host–pathogen dynamic interactions. Significantly, the advantages offered by ImFC support its diagnostic potential, with a prospect for translation into clinical practice [56]. Recent advancements in imaging flow cytometry have significantly improved its detection performance, offering an enhanced sensitivity, wavelength range, throughput, and detection limits, especially in fluorescence performance. A study by Luo et al. demonstrated a deep-learning-enabled imaging flow cytometry system for the high-throughput detection of Cryptosporidium and Giardia in drinking water. This system achieved a classification accuracy greater than 99.6%, with a sensitivity of 97.37% and a specificity of 99.95%, showcasing the system’s high-speed analysis capability at 346 frames per second [57]. The capacity to analyze microbial populations, as well as to scrutinize host–pathogen interactions, coupled with ImFC’s potential diagnostic capabilities, underscore ImFC’s immense potential for integration into clinical practice, which could revolutionize patient care and infectious disease management.

ImFC offers the capability for cell sorting, a feature available in specialized FC sorters [43,44]. ImFC offers a single-cell resolution and highly accurate label-free sorting, above 90%, in several experimental conditions [58]. Label-free cells are easier to manipulate thereafter and do not need to undergo the preparation step of labeling that may lead to a decrease in viability. The increased accuracy of ImFC in isolating single target cells is believed to be mostly based on the capability for morphological data analysis [58]. The implementation of machine learning algorithms is considered to further assist in the development of highly accurate, label-free cell sorting [59,60]. The unique capability of ImFC to perform cell sorting at a single-cell resolution, with an impressive accuracy of above 90% in diverse experimental conditions, opens up new frontiers in cell-based research. Notably, its ability to perform label-free sorting, enhanced by morphological data analysis and further augmented with machine learning algorithms, minimizes the need for preparation steps that could potentially reduce cell viability, thereby simplifying cell manipulation and ensuring a higher degree of cell integrity.

It is important to mention a recent and significant stride forward in the field. This is the introduction of the FACSDiscover S8 Cell Sorter (BD Biosciences), which is the first commercial imaging sorter, combining spectral and morphological image-based sorting capabilities [61,62]. Similarly, Visionsort (Thinkcyte) utilizes ghost cytometry and AI-powered algorithms to offer robust and accurate sorting capabilities [63,64].

ImFC has made significant strides since its inception, particularly with the introduction of commercial systems like Imagestream and FlowSight. Based on these advancements, ImFC has been applied in the label-free analysis of cell cycle distribution and cellular processes such as polarized antigen distribution in B cells during an immune response. ImFC has also been proven invaluable in studying intracellular pathogens, such as Toxoplasma gondii and Mycobacterium tuberculosis infections, thus indicating a promising avenue for the integration of this technology into clinical practice. Another important feature of ImFC is its cell sorting capability, providing highly accurate sorting at a single-cell resolution. The implementation of machine learning algorithms has further refined this process, reducing the need for preparation steps that could compromise cell viability. Collectively, the progress in the field underscores the significant potential of ImFC for advancing cell-based and clinical research, thereby contributing substantially to the medical and scientific industries.

## 4. Implementation of Machine Learning and Artificial Intelligence

Artificial intelligence (AI) and machine learning (ML) represent transformative technologies that are revolutionizing various sectors of society. AI refers to the simulation of human intelligence processes by machines, especially computer systems, while ML, a subset of AI, involves the development of algorithms that allow computers to learn and make decisions from data, thus improving their performance over time without being explicitly programmed. The application of ML algorithms has been widely used in imaging flow cytometry [64]. In a multiplex analysis by Eulenberg et al., deep learning offered the prospect of reconstructing biological processes such as cell cycle distribution, as well as disease progression [65]. Importantly, deep-learning-based predictions, apart from being sensitive, have been proven fast enough to support the high-throughput of generated data from ImFC. A deep learning approach has been successfully used to assess imaging flow cytometry data and is believed to have changed the landscape of high-throughput cell analysis [66]. Several successful applications support this notion. For example, 3D imaging flow cytometry (3D-ImFC) was performed by Subramanian R et al. to reveal hepatic stellate cell (HSC) and liver endothelial cell (LEC) morphology at a single-cell resolution. In their study, a combination of transmission and side-scattered single-cell images of liver cells with artificial intelligence was proposed to provide a staging system of NASH progression [67]. Furthermore, tomographic imaging flow cytometry (tIFC) has arisen to prevail over 2D to the 3D imaging of the surfaces and internal structures of particles [68]. The successful integration of AI and ML in imaging flow cytometry exemplifies the exciting prospects of these technologies in advancing our understanding of cell biology and disease mechanisms. Importantly, ImFC, through the adoption of microfluidics and lab-on-chip technologies, aims to simplify and reduce the costs of these analyses, with high-resolution single-cell imaging maximizing data extraction and enabling the use of smaller sample sizes. The anticipated benefits of integrating machine learning and deep learning into ImFC are substantial and expected to drive future innovations in the field [69].

ImFC has proven to be a valuable tool in the field of protein therapeutics, specifically, it was successfully exploited in a study of protein–silicon oil (SO) complexes. These complexes arise when protein particles adsorb onto silicon oil droplets, a process which has the potential to elicit an immune response, thus making it crucial to understand and monitor. In the study by Probst et al., ImFC provided a unique ability to measure the key parameters of these insoluble particles, including their number and shape. To further enhance the utility of this technology, researchers have incorporated ML techniques. By using a high amount of particle image data generated by ImFC, they have developed an ML model that can effectively categorize and count protein–SO compounds [70]. This combination of imaging flow cytometry and machine learning not only enhances the analysis of protein–SO complexes, but also broadens the scope of future applications in protein therapeutics and other related fields.

Equally prestigious is the contribution of ImFC and artificial intelligence to the classification of non-white blood (non-WBC) cells such as circulating rare cells in peripheral blood nucleated cells (PBNCs). Hirotsu et al. combined label-free ImFC with artificial intelligence software and managed to distinguish circulating rare cells as non-WBC fractions among PBNCs in cancer patients and healthy volunteers. Their described method offers exciting prospects for cancer patient monitoring and therapy optimization [71].

In a recent publication [72], Doan et al. detailed a process for analyzing ImFC images through deep learning techniques: by initially training a model with sample images possessing a specific phenotype, the model then uses the image pixels as inputs to forecast outcomes for new images. This direct analysis method was similarly employed in a recent investigation using traditional microscopy, where a fluorescent differentiation marker served as the factual basis for training a classifier. This classifier was then able to predict cell differentiation proactively based on the bright-field images [73].

Deep cytometry is a recent implementation of deep learning, in which image reconstruction is not necessary and real-time, non-supervised cell sorting can be achieved [74]. The future implications of deep cytometry are beginning to emerge. First, by employing deep learning algorithms, cell analysis and sorting can be performed in real time with minimal human intervention. This can dramatically increase the speed and efficiency of the process, enabling the handling of larger samples and potentially leading to more robust results. Second, AI could potentially be used to identify complex patterns or characteristics in cells that may not be immediately apparent to human analysts, based on subtle correlations and features. Third, AI holds promise in predicting future trends or behaviors based on historical data. This could be instrumental in studying disease progression or the effects of treatments at a cellular level. By analyzing past cytometry data using AI, predictive models can be created to forecast cellular responses under certain conditions.

AI, ML, and especially deep learning techniques have had a transformative impact on imaging flow cytometry, enabling unprecedented accuracy and efficiency in multicellular data analysis and increasing our understanding of complex biological phenomena such as disease progression and immunity. AI and ML facilitate highly accurate, real-time, unsupervised cell analysis. They also hold the potential to provide valuable insights into complex cell morphology, opening new frontiers in cell-based research, and suggest future prospects for clinical application.

## 5. Imaging Flow Cytometry and Hematology: Fundamentals for a Paradigm Shift?

In the previous sections of this review, we revisited the concept that imaging flow cytometry represents a significant technological advancement in the field of cytometry by merging the high-throughput capabilities of flow analysis with the image-based features of microscopy. This innovative approach has the potential to open up new avenues for the study of hematological diseases, offering distinct advantages over traditional cytometry, as well as complementing and/or competing with other novel molecular methodologies such as next-generation sequencing (NGS).

NGS has revolutionized hematology, providing insights into the genetic and molecular underpinnings of various blood disorders [75,76]. NGS technologies have enabled the comprehensive genomic profiling of hematological malignancies, such as leukemia and lymphoma, revealing the complex mutational landscapes that drive these diseases [77,78,79,80]. This has not only improved our understanding of disease pathogenesis, but has also facilitated the development of targeted therapies, leading to more personalized and effective treatment strategies, and has been instrumental in the detection of measurable residual disease (MRD), a critical factor in patient prognosis and treatment success [81,82,83,84]. Despite the challenges associated with data interpretation and integration into clinical practice, the impact of NGS in hematology has been transformative, and its potential for further advancements in diagnosis, prognosis, and treatment continues to be immense.

NGS and traditional flow cytometry, while distinct in their methodologies, both play crucial roles in the field of hematology. They complement each other in that flow cytometry provides the rapid, real-time analysis of cellular characteristics and protein expression, while NGS offers a deep dive into the genetic and molecular landscape of cells. However, they also compete in certain areas. For instance, while flow cytometry has been the gold standard for immunophenotyping, cell activity status, and measurable residual disease detection, NGS is increasingly being used for such applications, since it also offers a great sensitivity and specificity in detecting disease-associated genes and low-frequency mutations [85,86,87,88,89].

Beyond classic cytometry, one of the primary advantages of ImFC is its high-throughput capabilities. Unlike fluorescence microscopy, which is limited to processing a large number of cells, and NGS, which requires extensive time for sequencing and data analysis, ImFC can rapidly process and analyze up to 5000 cells/objects per second. This high-throughput capability allows for a more comprehensive analysis of cell populations, which is particularly beneficial in the context of hematological diseases, where disease progression can be monitored by changes in cell populations. In addition to its high-throughput capabilities, ImFC provides detailed morphological information about the cells it analyzes, including cell size, shape, internal complexity, and the distribution of specific markers in the cell. This level of detail is not typically available in NGS, which focuses more on genetic and molecular information. In hematological diseases, cellular morphology can provide important diagnostic and prognostic information, making ImFC a valuable tool in these contexts [17,90]. ImFC also leverages the power of deep learning for data analysis. Deep-learning-based predictions, apart from being sensitive, have been proven fast enough to support the high throughput of data generated from ImFC. This combination of ImFC and artificial intelligence can provide a more efficient and streamlined analysis compared to NGS, which often requires more complex and time-consuming data analysis. Novel developments, such as virtual freezing imaging flow cytometry, suggest that the implementation of information-rich cell data with artificial intelligence algorithms can further improve ImFC’s output and throughput [91].

Kalfa et al. described the use of ImFC to enhance the study of erythroid maturation, a process traditionally observed through microscopy, by combining the quantitative analysis capabilities of flow cytometry with the morphological assessment power of microscopy. ImFC is presented as a powerful tool for enumerating the various stages of erythropoiesis from primary tissue and for culturing progenitors to study rare enucleating cells, bridging the gap between microscopy and flow cytometry to offer comprehensive insights into erythropoiesis [92].

Fuller et al. validated the effectiveness of an advanced immuno-flowFISH method, combining immunophenotyping and FISH within an automated imaging flow cytometry framework to accurately detect chromosomal abnormalities in CLL, achieving a sensitivity surpassing that of current clinical standards. The integration of high-throughput imaging flow cytometry with specific fluorescent probes and immunophenotypic markers allowed for the precise identification of CLL cells and their chromosomal defects, offering substantial improvements in the detection limits for minimal residual disease monitoring. Technically, the process involved cell staining with specific fluorophore-conjugated antibodies, DNA denaturation, and hybridization with FISH probes, followed by automated, high-resolution image acquisition and analysis, enabling the simultaneous assessment of multiple chromosomal abnormalities in a single test without prior cell separation [93]. Similarly, Tsukamoto et al. introduced Immunophenotyped-Suspension-Multiplex (ISM)-FISH, a novel diagnostic approach for the simultaneous detection of the critical chromosomal translocations (t(4;14), t(14;16), and t(11;14)) in multiple myeloma, using immunophenotyping with anti-CD138 antibody and multiplex fluorescence in situ hybridization (FISH) analyzed via imaging flow cytometry. This method surpasses traditional FISH in sensitivity and efficiency, enabling the analysis of over 25,000 nucleated cells with a detection sensitivity of up to 0.1%, demonstrating a high concordance with standard FISH and offering a rapid, sensitive, and reliable diagnostic tool for guiding individualized treatment strategies in multiple myeloma [94].

In conclusion, while NGS has made significant contributions to our understanding of hematological diseases at a molecular level, ImFC offers unique advantages in terms of its high-throughput capabilities, detailed morphological information, deep learning integration, and label-free sorting. As such, ImFC represents a powerful tool in the study of hematological diseases and holds great promise for the future of diagnostics and research in this field.

## 6. Future Perspectives

ImFC is a relatively new technology in the field of cytometry. However, new ImFC systems are currently under development that have increased its speed and analytical power [45,95]. A stimulating example of ImFC development is ghost cytometry, described by Ota et al. as an image-free rapid fluorescence “imaging” cytometry which implements a single-pixel detector of spatial information obtained from the motion of cells relative to a static optical structure. Such data computationally reconstruct cell morphology and allow for rapid, accurate, and inexpensive analysis [46,47], as well as the potential for cell sorting [48]. Recent advancements in ghost cytometry are supported by a conference presentation and recent preprint, which provide evidence for the utility of the methodology and its application in the detection of acute leukemia cells, respectively [96,97]. Additional larger studies by diverse research groups will be needed to verify these promising results.

Suzuki et al. introduced label-free chemical imaging flow cytometry, merging the rapid analysis capabilities of flow cytometry with the specificity of fluorescence imaging and digital analysis, enhanced by a novel pulse pair-resolved, wavelength-switchable Stokes laser. This advancement allowed for the fastest multicolor stimulated Raman scattering (SRS) microscopy of cells in flow, achieving an unprecedented throughput of up to ∼140 cells/s on a 3D acoustic focusing microfluidic chip. Demonstrating its versatility, the method was applied to study metabolic heterogeneity in microalgae and to detect cancer in blood without the need for markers, leveraging deep learning for enhanced accuracy [98].

## 7. Conclusions

Imaging flow cytometry (ImFC) is a technology that has gained ground during recent years based on its potential in both research and clinical settings, and promise for revolutionizing our ability to study, quantify, depict, and ultimately understand cellular mechanisms. In the field of basic and translational research, ImFC provides information about cellular morphology in addition to the high-throughput capabilities of a flow cytometer, illuminating our understanding of cellular structures and their respective functions. By allowing for the high-throughput analysis of individual cells in a population, it offers a detailed and objective view of cellular dynamics, enabling researchers to observe processes like cell division, antigen distribution, and intracellular pathogen behavior in real time. The additional layer of morphological data greatly aids in the study of complex biological phenomena and can significantly enhance accuracy. In the clinical setting, even though current data are limited, the high precision of ImFC and its ability to perform label-free sorting, as well as its compatibility with machine learning algorithms, indicate a promising future potential for diagnostic applications. From studying disease progression at the cellular level to potentially tailoring patient-specific treatments, ImFC might improve patient care, given that it is successfully implemented. Its capacity for the real-time analysis of large cell populations also holds promise for implementation in high-throughput clinical workflows, potentially leading to quicker diagnoses and treatment. In conclusion, the impact of ImFC in both research and clinical environments signals a promising future for cell-based studies and medical practices.

## Figures and Tables

**Table 1:** Applications of imaging flow cytometry.

Application	Description	Ref.*
Spatiotemporal calcium mobilization	ImFC has been used for the analysis of calcium ion (Ca^2+^) mobilization in T cells, combining the statistical rigor of conventional flow cytometry with microscopic spatial information to observe Ca^2+^ flux in response to stimuli.	[28]
Virus–host interactions	The imaging capabilities of ImFC have been exploited to analyze viral infection stages and virus host–interactions. Analyzed viruses include mimivirus, respiratory syncytial virus, and human immunodeficiency virus.	[29,30]
Extracellular vesicles (EVs) and exosomes analysis	The applications of advanced ImFC enable detailed analyses of EV subset composition, the identification of exosomes in the circulation and their tissues of origin, and the determination of their functional immunological impact and both physiology and pathology (such as cancer).	[31,32]
Quantification of protein–protein interactions in rare cell populations	Proximity ligation imaging cytometry (PLIC), designed to address the challenges of proteomic analysis in rare cell populations, has been applied to medullary thymic epithelial cells and allowed for the high-resolution detection/quantification of protein–protein interactions and post-translational modifications at the single-cell level.	[33]
Quantification of senescent cells	ImFC has allowed the simple, rapid, and quantitative detection of senescent cell populations. This detection included live cells and required no staining.	[34]
Golgi fragmentation analysis	ImFC can be used for quantifying Golgi fragmentation, offering a rapid, automated, and unbiased method capable of analyzing over 50,000 cells per sample. The technique has proven robust for future Golgi dynamics research.	[35,36]
Bacterial phenotypes and interactions	ImFC has been successfully applied in the analysis of the morphological characteristics of bacterial cells, as well as the interactions between different bacteria and with the host cells.	[37,38,39]
Mitochondrial dynamics	ImFC introduces a novel, unbiased, and high-throughput approach for measuring mitochondrial fusion activity using the Amnis ImagestreamX™ MKII and IDEAS™ V6.1 software. This method enhances the traditional polyethylene glycol (PEG) fusion assay by efficiently detecting and analyzing fused cells—identified by their dual nuclei and the co-localization of different mitochondrially targeted proteins.	[40]
Label-free analysis of cell cycle distribution	ImFC has been used to demonstrate a polarized antigen distribution in B cells during an immune response that was sustained among progeny. This successfully revealed the cell cycle distribution of cells and a consistent pattern of polarized antigen distribution in B cells during immune responses, a pattern that persists across generations. Imagestream X ImFC platform and IDEAtool v6.1 software were used for acquisition, while cellprofiler was used for further analysis.	[41]
Analysis of intracellular pathogens	ImFC has successfully been used for the analysis of Toxoplasma gondii and Mycobacterium tuberculosis infections in cell lines. This type of analysis offers a prospect for studying host–pathogen dynamic interactions. IDEAS software with the Feature Finder algorithm was implemented.	[42]
Cell sorting	ImFC offers the capability for cell sorting, a feature available in specialized FC sorters. ImFC offers single-cell resolution and highly accurate label-free sorting, above 90%, in several experimental conditions. An on-chip sorting technology was developed using nanofluidics and electrostatic force, implementing a phase-contrast/fluorescence microscope ImFC system.	[43,44]
Microparticle imaging	ImFC has been used for high-throughput single-microparticle imaging flow analyses. The authors developed a rapid optical iMFC platform that contained self-focusing microfluidic apparatus, optoelectronic communication, and an informatics analysis system.	[45]
Ghost cytometry	Ghost cytometry, a technique for classifying cells and other microparticles without the need for labeling or imaging, has been used in conjunction with ImFC. It offers the potential for cell sorting. Ghost cytometry was developed as an image-free fluorescence cytometry utilizing a single-pixel detector, which compressively translates spatial information from cell movement across a static, randomly patterned optical structure into sequential signals.	[46,47,48]

* Relevant references.

## Data Availability

Not applicable.
